# Cardiac MRI and FDG-PET in the diagnosis of cardiac sarcoidosis

**DOI:** 10.1186/1532-429X-16-S1-P299

**Published:** 2014-01-16

**Authors:** Richard Coulden, Hefin Jones, Emer Sonnex, Indrajeet Das, Jonathan Abele

**Affiliations:** 1 Dept Radiology & Diagnostic Imaging, University of Alberta Hospital, Edmonton, Alberta, Canada

## Background

Sarcoidosis is a multisystem disorder with cardiac involvement in 25% of cases [1]. Diagnosis of cardiac sarcoidosis is challenging with FDG-PET and cardiac MRI (CMRI) proving most reliable. We compare FDG-PET and CMRI with delayed enhancement (LGE) in patients with biopsy proven extra-cardiac sarcoidosis being investigated for cardiac involvement.

## Methods

30 patients meeting Japanese Ministry of Health & Welfare guidelines [2] for clinical cardiac sarcoidosis were investigated with FDG-PET CT (Gemini TF Philips) and CMRI (Aera 1.5T Siemens) on the same day. Patients undergoing FDG-PET followed a 24 hour low-carbohydrate diet and overnight fast [3]. CMRI examination included SSFP assessment of left ventricular (LV) function, short axis T2-weighted STIR and PSIR-LGE 10 minutes post 0.2 mmol/kg GdDTPA. Images were reviewed by experienced readers blinded to the results of the other examination. FDG-PET was considered positive if any segment (AHA 17 segment model) had an SUVmax > 3.6 (3). CMRI was considered positive if any segment showed 'sarcoid-type' LGE. In no case was edema present on STIR imaging without LGE in the same segment on subsequent PSIR.

## Results

**Figure 1:**
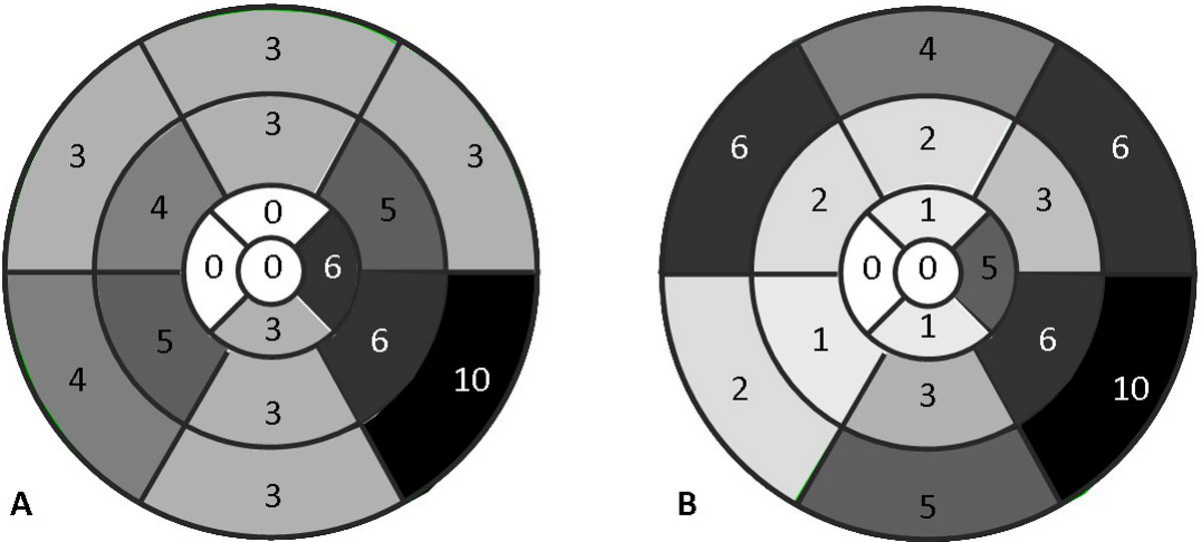
**LV distribution of LGE (A) and significant myocardial FDG uptake (B) for all 30 patients**. By both modalities, cardiac sarcoidosis predominantly involves the lateral wall with relative sparing of the distal anterior wall, distal septum and apex.

FDG-PET and CMRI were positive in 10 patients; FDG-PET +ve and CMRI -ve in 3; FDG-PET -ve and CMRI +ve in 4; both -ve in 13. Distributions of FDG and LGE throughout the myocardium are summarized in Figure 1. In 2 cases where FDG and CMRI were +ve, LGE was in an ischemic pattern and both patients had known recent iscemic events (sub-endocardial). 6 of the remaining 8 had impaired LV function (EF < 50%). All FDG +ve/CMRI -ve cases had intense mediastinal/lung FDG uptake. All 4 FDG -ve/CMRI +ve had either long-standing sarcoidosis ( > 2 years) or absent node/lung FDG activity. Seven of the cases, -ve by both modalities, had no significant or minimal node/lung FDG uptake (SUVmax < 2.5).

## Conclusions

Previous single modality studies have suggested sensitivity and specificity for FDG-PET of 89% and 78% and CMRI of 75% and 77% respectively. In our study, 43% showed no cardiac sarcoidosis by either modality and half of these had minimal or no FDG-PET evidence of active sarcoidosis elsewhere. Are these false -ve FDG-PET/CMRI studies or are the clinical criteria too sensitive? Our findings suggest FDG-PET and CMRI-LGE show different degrees of cardiac sarcoid involvement: FDG-PET indicating active inflammatory disease, LGE showing severe edema and scar. Those patients with LGE and no myocardial FDG uptake appear to have 'burnt-out' disease.

## Funding

None.
